# Exclusive Production of Gentamicin C1a from *Micromonospora purpurea* by Metabolic Engineering

**DOI:** 10.3390/antibiotics8040267

**Published:** 2019-12-14

**Authors:** Zeng Wei, Xianai Shi, Rong Lian, Weibin Wang, Wenrong Hong, Shaobin Guo

**Affiliations:** College of Biological Science and Engineering, Fuzhou University, Fuzhou 350108, China; zengwei0331@163.com (Z.W.); shixa@fzu.edu.cn (X.S.); lianr@amoydx.com (R.L.); N180820026@fzu.edu.cn (W.W.)

**Keywords:** *Micromonospora purpurea*, gentamicin C1a, N-methyltransferase, metabolic engineering

## Abstract

Gentamicin C1a is an important precursor to the synthesis of etimicin, a potent antibiotic. Wild type *Micromonospora purpurea* Gb1008 produces gentamicin C1a, besides four other gentamicin C components: C1, C2, C2a, and C2b. While the previously reported engineered strain *M. purpurea* GK1101 can produce relatively high titers of C1a by blocking the *genK* pathway, a small amount of undesirable C2b is still being synthesized in cells. Gene *genL* (*orf6255*) is reported to be responsible for converting C1a to C2b and C2 to C1 in *Micromonospora echinospora* ATCC15835. In this work, we identify the *genL* that is also responsible for the same methylation in *Micromonospora purpurea*. Based on *M. purpurea* GK1101, we construct a new strain with *genL* inactivated and show that no C2b is produced in this strain. Therefore, we successfully engineer a strain of *M. purpurea* that solely produces gentamicin C1a. This strain can potentially be used in the industrial production of C1a for the synthesis of etimicin.

## 1. Introduction

**Figure 1:**
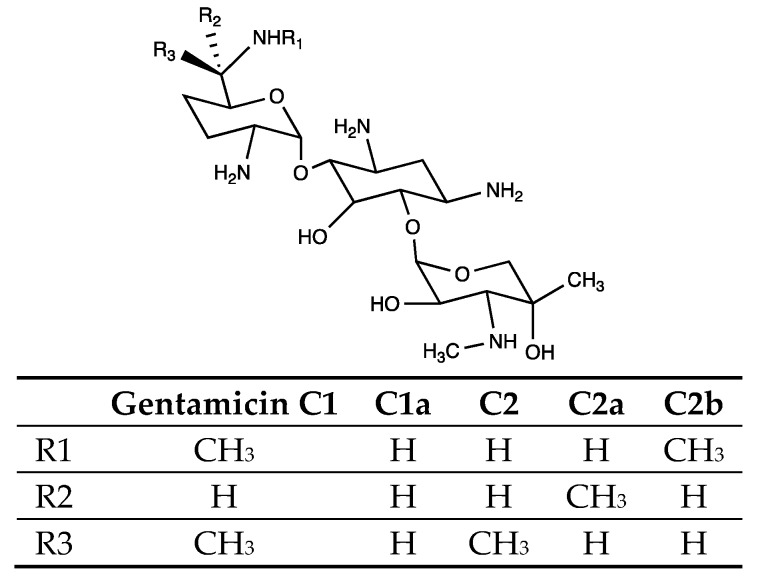
General structure of gentamicin C components. There are mainly five variants of gentamicin C: C1, C1a, C2, C2a, and C2b. They differ from each other at the three R sites annotated in this structure. The corresponding groups at each R site of these five components are listed in the table.

Gentamicin C is a broad-spectrum aminoglycoside and water-soluble antibiotic, and an important anti-infective drug in clinical uses. Gentamicin C is produced in a genus of bacteria called *Micromonospora* [1]. Currently, the engineered strain *Micromonospora purpurea* Gb1008 series are mainly used in the industrial production of gentamicin C. Gentamicin C mainly consists of five clinically essential components: C1, C1a, C2, C2a, and C2b (Figure 1) [2]. The five gentamicin components differ from each other mainly at the methylation modification sites. Methylation plays a significant role in the antimicrobial activity of gentamicin C. Among the four methylation sites, C-6’ and 6’-N methylations are of the most significance [3]. Previous studies demonstrate that gentamicin C1a shows minimal cochlear effects, and is the most effective antibiotic compared to the other four components [4]. However, at the beginning of the 21st century, the therapeutic utility of aminoglycosides was being threatened by the emergence of resistance traits among the clinical isolates, which once caused panic in public [5,6]. Etimicin, the fourth generation of the new semi-synthetic aminoglycoside antibiotics [7], is considered to be useful in the treatment of aminoglycoside-resistant bacterial infections [8], and has been widely used as a human and veterinary drug against Gram-positive and Gram-negative bacterial infections. Etimicin is a 1-N-ethyl derivative of gentamicin C1a [9], and it has many advantages over traditional aminoglycoside antibiotics: a broad antimicrobial spectrum, stronger antimicrobial activity, lower ototoxicity, and nephrotoxicity [10]. However, the industrial production of gentamicin C1a, the synthetic precursor of etimicin, is still challenging. Gentamicin C1a has to be separated from other components of gentamicin C components, which holds back the large-scale production of etimicin.

**Figure 2:**
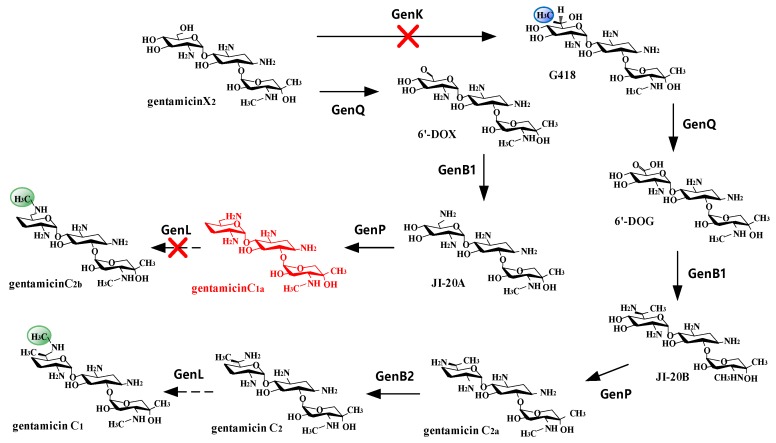
Biosynthesis pathway of gentamicin C complex in *M. purpurea*. There are two main pathways starting from gentamicin X2: (1) GenK→GenQ→GenB1→GenP→GenB2→GenL pathway produces gentamicin C2a, C2, and C1; (2) GenQ→GenB1→GenP→GenL pathway produces gentamicin C1a and C2b. By knocking out both GenK and GenL, a strain exclusively producing gentamicin C1a can be obtained, as shown in the figure.

Most of the key steps in the biosynthesis of gentamicin C components have been elucidated in previous work (Figure 2): *genK* is an S-Adenosyl methionine (SAM) dependent methylation gene and is responsible for C-6’ methylation [11,12]; *genP* is thought to be responsible for 3’, 4’ double dehydroxylation [13,14]; *genQ* is related to Gentamicin C-6’ dehydrogenation [15]; *genB1* catalyzes the transformation of G418 to JI-20B and X2 to JI-20A [16]; *genB2* involves in the epimerization of gentamicin C2 and C2a in vitro [16]; and *genL* is reported to be responsible for converting C1a to C2b and C2 to C1 of *Micromonospora echinospora* ATCC15835 [17].

Previously, our team constructed an *M. purpurea* strain GK1101 to increase the titers of C1a [11], where C1 metabolic flux was blocked by inactivating the *genK* pathway (Figure 2). However, this strain still produced a small amount of gentamicin C2b and an extra separation step is required to meet the requirements of industrial production of C1a. In this study, we perform gene knockouts in the genome of *M. purpurea* GK1101. We confirm that the *genL* of *M. purpurea* is responsible for the methylation of C2 to C1 and C1a to C2b, as no C1 nor C2b can be detected in the knockout strains, the same as in *M. echinospora*. By combining the GK1101 strain and *orf6255* gene knockout, we are able to obtain the engineered strain that only produces gentamicin C1a. To our best knowledge, this is the first strain that solely produces C1a component. This strain should provide a solid foundation for the preparation of etimicin.

## 2. Material and Methods

### 2.1. Strains and Plasmids

All bacterial strains and plasmids used in this study are listed in Table 1, *M. purpurea* Gb1008, GK1101, *Escherichia coli* ET12567 (pUZ8002), and shuttle plasmid pKC1139 are preserved in our laboratory. *E. coli* Top10 was purchased from Thermo Fisher Scientific. The cloning vector pMD19-T was purchased from TaKaRa. Luria-Bertani (LB) broth (Sangon Biotech, Shanghai, China) was used for the *E. coli* culture. Mannitol soya flour medium (MS) was used for the conjugation [18]. The previously described media and culture conditions were used for gentamicin production [11]. Antibiotic microbial verification culture medium I: dipotassium phosphate 0.3%, beef cream 0.3%, tryptone 1%, agar powder 2%. The final concentrations of the antibiotics in the LB medium were as follows: ampicillin, 100 μg/mL; apramycin, 50 μg/mL. In the MS medium, the final concentrations of both apramycin and nalidixic acid were 25 μg/mL.

### 2.2. Construction of the Disruption Plasmid

DNA isolation and manipulation were performed as described by Sambrook [19]. The *genL* (*orf6255*) gene was analyzed using NCBI database. Using *genL* upstream and downstream sequences as homologous exchange arms, two pairs of primers, L1/L2 and L3/L4 (sequences listed in Table 2), were used to amplify upstream and downstream exchange arms. Target bands of PCR samples were recovered using gel extraction. The upstream exchange arm was digested by *Eco*RI and *Xba*I and the downstream exchange arm was digested by *Xba*I and *Hin*dIII, reclaiming the short fragments. The thermosensitive plasmid pKC1139 was digested by *Hin*dIII and *Eco*RI. Three fragments were ligated and transformed into *E. coli* Top10 competent cells. Positive clones were screened and homologous recombinant plasmid pKTL12 was obtained. According to the principle of homologous recombination, a pair of primers L5/L6 were designed for screening and identifying single or double exchange. The primer sequences and its restriction enzymes are shown in Table 2.

### 2.3. Construction of the Disruption Strain

The recombinant plasmid pKTL12 was transformed into *E. coli* ET12567, and introduced into *M. purpurea* GK1101 (Δ*genK*) using the conjugative transfer on MS medium at 37 °C for 16 h. After overlaying with medium containing apramycin and nalidixic acid, the incubation was continued at 37 °C for 5 days. Single and double crossover mutants were then selected and screened as previously described [20]. To check whether a double crossover event had occurred, we designed primers N5/N6 to screen single and double crossover mutants. We selected the ampicillin-sensitive colonies and lysed them to obtain genomic DNAs, which were used as templates to amplify using primers N5/N6 to check whether *genL* was successfully deleted from the genome. Sequencing was also used to further verify the results. The single crossover mutant was named GbKL201 and the double crossover was named GbKL202. The same method was used to construct GbL201 and GbL202.

### 2.4. Antibiotic Separation and Analysis

The fermentation process and metabolite extraction of *M. purpurea* was done based on previously described protocols [21]. Its antibiotic activity was detected by the cylinder-plate method, which was done according to Pharmacopoeia of the People’s Republic of China (2010 edition) [22]. The components of the product were analyzed by thin layer chromatography (TLC) using silica gel GF254 plate. The development solvent used in the assay was the underlayer of the mixture (chloroform: methanol: ammonia in 1:1:1 volume ratio). Accurate component ascertainment was performed by electrospray ionization mass spectrometry (ESI/MS) using Agilent 6520 quadrupole-time-of-flight mass spectrometer. The scanning range of Q-TOP-MS is set as following: Positive ion mode *m*/*z* 100~800; the dry gas is N_2_; the flow rate is 8mL/min; the temperature is 350 °C; atomization pressure is 2.07 × 10^5^ Pa; capillary voltage is 3500 V; the fragmentation voltage is 135 V; and Agilent MassHunter is used to analysis MS data (B.04.00).

### 2.5. Measurement of Titers of Gentamicin

A microbiological assay was used to measure the titers of gentamicin. Concentrations of gentamicin in supernatants of the fermentation broth were measured by the agar diffusion method. The 24 h cell culture of *Bacillus pumilus* was made into a suspension using sterile saline solution. Commercial gentamicin was diluted into 5 unit/mL (u/mL) and 10 u/mL using 0.3% potassium phosphate buffer. Titers of supernatants were estimated and supernatants were diluted into 5 u/mL and 10 u/mL. Plates were made with 20 mL of the antibiotic microbial verification media (listed in Section 2.1). Four oxford cups were set on each plate. The bacterial suspension was mixed with the same media and then added 5 mL on each plate as the top layer. Diluted gentamicin standard and supernatants were added to one of the four spots on plates and grown at 37 °C overnight. Measure the diameters of the bacteria-free zones and calculate the titers using the following equation:

$$\theta ={\mathrm{lg}}^{-1}\left[\frac{\sum T_{2}+\sum T_{1}-\sum S_{2}-\sum S_{1}}{\sum T_{2}+\sum S_{2}-\sum T_{1}-\sum S_{1}}\times I\right]$$

*θ*: Ratio of actual titer over estimated titer;
*S*_1_: Diameter of clear zones with 5 u/mL gentamicin;
*S*_2_: Diameter of clear zones with 10 u/mL gentamicin;
*T*_1_: Diameter of clear zones with estimated 5 u/mL supernatant;
*T*_2_: Diameter of clear zones with estimated 10 u/mL supernatant;
I: I = 0.301

Actual titer of supernatants = θ*estimated titer.

## 3. Results

### 3.1. Construction of the Disruption Strain

**Figure 3:**
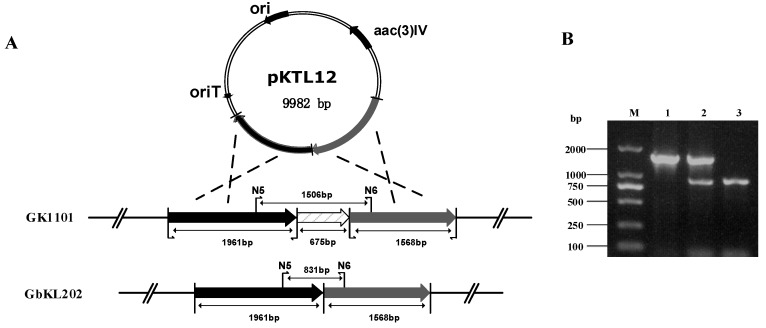
Targeted gene disruption in *M. purpurea* GK1101: (**A**) diagram showing the targeted gene area in GK1101 (starting strain) and GbKL202 (engineered strain); (**B**) PCR analysis of the genomic DNA from *M. purpurea* GK1101 and GbKL202 using primers N5/N6. The 1506 bp sequence corresponds to the intact *genL* gene in the *GK1101* (lane 1), the 1506 bp and 831 bp band in single crossover mutant strain (lane 2) and the 831 bp band (deleting 675 bp of *genL*) in GbKL202 (lane 3). The DL2000 DNA marker is in lane M.

We used NCBI BLASTN to analyze *genL* (*orf6255*), and the results indicate that it contains a SAM-binding domain. Enzymes containing this domain can potentially methylate different substrates, such as small molecules, lipids, nucleic acids, etc. (Figure S1, Table S1). To verify the function of the *genL* in *M. purpurea* and obtain C1a-producing strain, the disruption plasmid pKTL12 (using for deleting *genL*) was individually introduced into *M. purpurea* GbK1101 (g*enK*) by conjugation transfer. As a result, we deleted 675bp of *genL* in GbK1101 (Figure 3A). PCR verification was carried out using primers N5/N6. The amplified product of the double crossover mutant was compared with the PCR product from the wild-type and single crossover mutant. As we can see in Figure 3B, the PCR product of the double crossover mutant has only one band at 831 bp, where negative control has one band at 1506 bp and single crossover mutant has two bands at 1506 bp and 831 bp. Thus we obtain a double crossover mutant strain named GbKL202 (Δ*genK* Δ*genL*).

### 3.2. Analysis of the Metabolites

**Figure 4:**
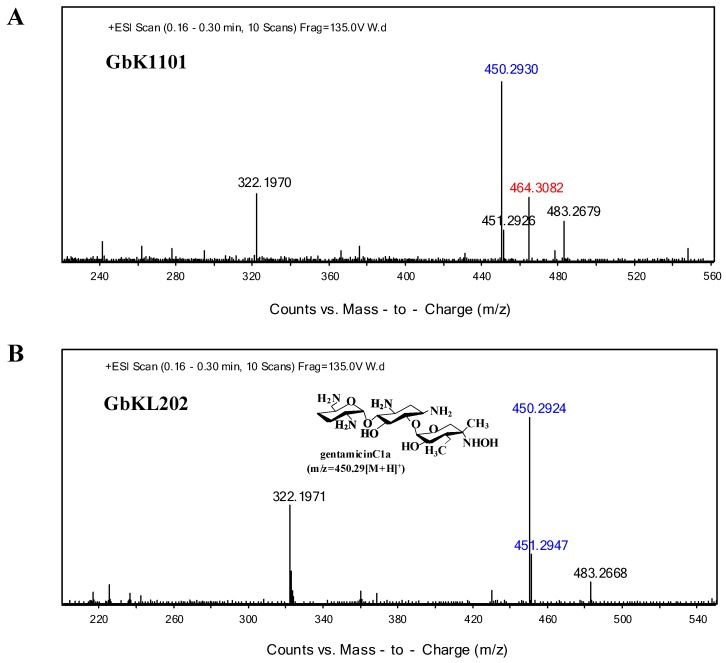
Metabolites of the disruption strain. (**A**) Mass spectrum analysis of metabolites from *M. purpurea* GK1101 (Δ*genK*). (**B**) Mass spectrum analysis of metabolites from GbKL202 (Δ*genK*Δ*genL*). In particular, the blue numerical value is gentamicin C1a (*m/z* = 450.2924[M + H] ^+^, *m/z* = 451.2947[M + D] ^+^), the green numerical value is gentamicin C2b (*m/z* = 464.30[M + H] ^+^).

We grew GbKL202 (experiment group) and GK1101 (control group). Metabolites from the fermentation broths of both strains were extracted. Thin layer chromatography (TLC) results showed that GbKL202 no longer produced the C2b component in comparison to GK1101 (Figure S2). Electrospray ionization mass spectrometry (ESI/MS) analysis revealed that metabolites from GbKL202 did not contain C2b (*m*/*z* = 464.3082 [M + H] ^+^), but contained a large amount of C1a (*m*/*z* = 450.2924 [M + H] ^+^, *m/z* = 451.2947 [M + D] ^+^) (Figure 4). In particular, the peak *m/z* = 322.197 [M + H]^+^ indicates a fragment ion peak formed as a result of Gentamicin C complex losing a purpurosamine ring [23]. Therefore, the results suggested that *genL* was a key gene for converting C1a to C2b by adding a methyl group at 6’-N. Then we compared the antibiotic titer of the starting strain and the engineered strain. The antibiotic titer of the starting strain GK1101 was 792.24 u/mL, and that of the engineered strain GbKL202 was 780.39 u/mL, indicating that they have comparable yields. In summary, we constructed a C1a-producing strain that does not produce other variants of gentamicin C.

### 3.3. genL is Involved in Converting C2 to C1 in M. Purpurea

**Figure 5:**
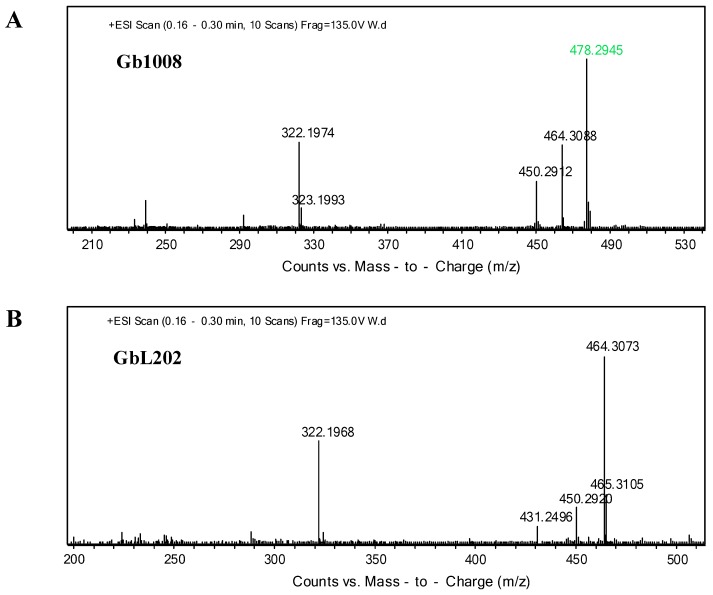
Metabolites of the disruption strain. (**A**) Mass spectrum analysis of metabolites from *M. purpurea* Gb1008. (**B**) Mass spectrum analysis of metabolites from GbL202 (Δ*genL*). Note that the purple numerical value is gentamicin C1 (*m/z* = 478.2945[M + H] ^+^).

According to the metabolic synthesis pathway of gentamicin, we notice that gentamicin C1 is obtained by methylation of C2 at the same 6’-N position. We suspected that *genL* was also the key gene responsible for converting C2 to C1 of *M. purpurea*. Based on this assumption, we deleted *genL* in the strain *M. purpurea* Gb1008 to obtain a new strain named GbL202 (Δ*genL*) (Figure 3A). TLC and ESI/MS results showed that metabolites from GbL202 did not contain gentamicin C1 (Figure S3 and Figure 5). We showed that *genL* was also responsible for converting C2 to C1, and the anaplerosis experiment of *genL* in GbHL202 confirmed this result (Figures S4 and S5).

## 4. Discussions

The gentamicin C pathway has been well studied. Researchers have identified most of the enzymes responsible for its biosynthesis (Figure 2). Among all these enzymes, methyltransferases determine the composition of the gentamicin C complex. There are two main pathway branches starting from the compound gentamicin X2. It can be dehydrogenated by GenQ directly; but it can also be dehydrogenated by GenQ after being methylated into an intermediate called G418 by GenK. The extra methyl group added by GenK differentiates gentamicin C1a from C2a. And then two more enzymes (GenB2 and GenL) are involved to add even more diversities with C1a and C2a: C1a methylated into C2b; C2a epimerized into C2, which can then be methylated into C1. By knocking out certain genes encoding for these enzymes, we are able to direct the flow of the biosynthesis pathway to make the most out of the limited resources. Wild type strain *M. purpurea* Gb1008 produces all five components of gentamicin C: C1, C1a, C2, C2a, and C2b; GK1101 (Δ*genK*) strain produces C1a and C2b; GbL202 (Δ*genL*) produces C1a, C2, and C2a; GbKL202 (Δ*genK*Δ*genL*) produces only C1a.

Gentamycin C1a is a synthetic precursor of etimicin, which is a semi-synthetic aminoglycoside drug. Production of gentamycin C1a with the wild type strain is not only low in yield but also high in cost, which cannot meet the requirements of industrialization. Therefore, it is essential to develop an engineered strain exclusively producing gentamicin C1a. *M. purpurea* GK1101 (Δ*genK*) produces gentamicin C1a, but also traceable amount of C2b, which adds to the cost and work by requiring an extra separation step. Gene *genL* is shown to be responsible for converting C1a to C2b and C2 to C1 in *M. echinospora* ATCC15835 [17]. We experimentally confirmed that *genL* of *M. purpurea* is also responsible for the methylation of C2 to C1 and C1a to C2b. Thus, we constructed an engineered strain GbKL202 by knocking out *genL* based on GK1101 and showed that this new strain only produced C1a. As a result, gentamicin C1a can be accumulated in large amounts and C2b is no longer produced.

In summary, we successfully construct a single-component engineered strain of *M. purpurea* producing gentamicin C1a with high titers, which provide an ideal platform for the industrial production of gentamicin C1a and etimicin.

## Figures and Tables

**Table 1:** Strains and plasmids used in this study.

Strains or Plasmids		Relevant Characteristic	Reference or Source
**Strains**	*M. purpurea* Gb1008	Wild-Type Strain	Our lab
	GK1101	*M. purpurea* Gb1008 with Δ*genK*	Our Lab
	*E. coli* Top10	F-, mcrAΔ(*mrr-hsd RMS-mcrBC*), Δ*(ara-leu)769*, *recA1*, φ80, *araΔ139 lacZ*Δ*M15* △l*acX74 galK galU*, *rps*, *nupG, (Strr) endA1*	Thermo Fisher
	*E. coli* ET12567/pUZ8002	*Nonmethylating E. coli, CmL ^R^*; *Tra+, OriTRK2 derivative, Kan ^R^*	Our lab
	*Bacillus pumilus*	Used as detecting bacteria	Our lab
	GbL201	Single crossover mutant of *M. purpurea* Gb1008, with plasmid pKTL12	This study
	GbL202	*M. purpurea* Gb1008 with *genL* (*orf6255*) disrupted	This study
	GbKL201	Single crossover mutant of GK1101, with plasmid pKTP12	This study
	GbKL202	GK1101 with *genL*(*orf6255*) disrupted	This study
Plasmids	pMD19-T	Amp ^R^, Cloning vector for *E. coli*	TaKaRa
	pKC1139	6.5 kb, *E. coli–Streptomyces* shutter vector, Streptomyces replicon pSG5 *ori* *E. coli* replicon pUC *oriT lacZa* MCS Rep ^ts^ Ap ^R^	Our lab
	pTL1	pMD19-T containing upstream fragments, Amp ^R^	This study
	pTL2	pMD19-T containing downstream fragments, Amp ^R^	This study
	pKTL12	pKC1139 containing upstream and downstream fragments, used for *genL* (*orf6255*) disruption, AP ^R^	This study

CmL ^R^, chloromycetin resistance; ts, temperature sensitive; Kan ^R^, kanamycin resistance; Amp ^R^, ampicillin resistance; AP ^R^, apramycin resistance; MCS, multiple cloning site; Repts, temperature-sensitive replicon.

**Table 2:** Primer used in this study.

Name	Sequence ^a^
L1	5′-TTAGAATTCAGCAGGCGGGCCTCGTCGAGAAAGCGTT-3’
L2	5’-GGTCTAGAGATCGGAGATGCTCAAGATGG-3’
L3	5’-TTTCTAGATCTACTCCGTCGGCGAGTCG-3’
L4	5′-GGGAAGCTTAAAGTGGGCGACCACCAAGCACAAGAAG-3’
L5	5′-TTCGAGATCGTCAAGTACCGGGTC-3’
L6	5’-GGATGATGATGGAGATGGGCTTCG-3’

^a^ Restriction enzyme site are indicated by single underlines.

## Supplementary Materials

The following are available online at https://www.mdpi.com/2079-6382/8/4/267/s1, Figure S1: Domain analysis of *genL* (*orf6255*), Figure S2: Thin layer chromatography analysis of metabolites of GbKL202; Figure S3: Thin layer chromatography analysis of metabolites from GbL202; Figure S4: Construction of GbHL202 (*genL* anaplerosis of GbL202) and confirmation; Figure S5: MS and TLC analysis of metabolites from GbHL202; Table S1: List of *genL* (*orf6255*) conserved domain hits.

## References

[1] Weinstein M.J., Luedemann G.M., Oden E.M., Wagman G.H., Rosselet J.P., Marquez J.A., Coniglio C.T., Charney W., Herzog H.L., Black J. (1963). Gentamicin, a new antibiotic complex from *micromonospora*. J. Med. Chem..

[2] Rodriquez M., Cretoso D.S., Euterpio M.A., Russo P., Crescenzi C., Aquino R.P. (2015). Fast determination of underivatized gentamicin C components and impurities by LC-MS using a porous graphitic carbon stationary phase. Anal. Bioanal. Chem..

[3] Bezdjian A., Mujica-Mota M.A., Devic S., Daniel S.J. (2015). The effect of radiotherapy on gentamicin ototoxicity: An animal model. Otolaryngol. Head Neck Surg..

[4] Kobayashi M., Sone M., Umemura M., Nabeshima T., Nakashima T., Hellstrom S. (2008). Comparisons of cochleotoxicity among three gentamicin compounds following intratympanic application. Acta Otolaryngol..

[5] Boehr D.D., Jenkins S.I., Wright G.D. (2003). The molecular basis of the expansive substrate specificity of the antibiotic resistance enzyme aminoglycoside acetyltransferase-6′-aminoglycoside phosphotransferase-2″. The role of ASP-99 as an active site base important for acetyl transfer. J. Biol. Chem..

[6] Boehr D.D., Daigle D.M., Wright G.D. (2004). Domain-domain interactions in the aminoglycoside antibiotic resistance enzyme AAC (6′)-APH (2″). Biochemistry.

[7] Fan J., Zhao M., Liu J. (1995). Aminoglycoside antibiotics 89-07: Semisythetic and structure measurement. Chin. J. Antibiot..

[8] Chaudhary M., Kesava N.G., Kumar S., Payasi A. (2012). Comparative antibacterial activity of a novel semisynthetic antibiotic: Etimicin sulphate and other aminoglycosides. World. J. Microbiol. Biotechnol..

[9] Wang Y., Zhang Z., Wu L., Zhang X., Wang H., Ye W., Li P. (2014). Isolation and structure characterization of related impurities in etimicin intermediate P1 by LC/ESI-MSn and NMR. J. Pharm. Biomed. Anal..

[10] Yoshizawa S., Fourmy D., Puglisi J.D. (1998). Structural origins of gentamicin antibiotic action. EMBO J..

[11] Hong W., Yan L. (2012). Identification of *gntK*, a gene required for the methylation of purpurosamine C-6′ in gentamicin biosynthesis. J. Gen. Appl. Microbiol..

[12] Kim H.J., McCarty R.M., Ogasawara Y., Liu Y.N., Mansoorabadi S.O., LeVieux J., Liu H.W. (2013). GenK-catalyzed C-6′ methylation in the biosynthesis of gentamicin: Isolation and characterization of a cobalamin-dependent radical SAM enzyme. J. Am. Chem. Soc..

[13] Shao L., Chen J., Wang C., Li J.A., Tang Y., Chen D., Liu W. (2013). Characterization of a key aminoglycoside phosphotransferase in gentamicin biosynthesis. Bioorg. Med. Chem. Lett..

[14] Gu Y., Ni X., Ren J., Gao H., Wang D., Xia H. (2015). Biosynthesis of Epimers C2 and C2a in the Gentamicin C Complex. Chembiochem.

[15] Ni X.P., Sun Z.P., Zhang H.Y., He H., Ji Z.X., Xia H.Z. (2014). Genetic engineering combined with random mutagenesis to enhance G418 production in *Micromonospora echinospora*. J. Ind. Microbiol. Biot..

[16] Guo J., Huang F., Huang C., Duan X., Jian X., Leeper F., Deng Z., Leadlay P.F., Sun Y. (2014). Specificity and promiscuity at the branch point in gentamicin biosynthesis. Chem. Biol..

[17] Li S., Guo J., Reva A., Huang F., Xiong B., Liu Y., Deng Z., Leadlay P.F., Sun Y. (2018). Methyltransferases of gentamicin biosynthesis. Proc. Natl. Acad. Sci. USA.

[18] Hobbs G., Frazer C.M., Gardner D.C.J., Cullum J.A., Oliver S.G. (1989). Dispersed growth of *Streptomyces* in liquid culture. Appl. Microbiol. Biotechnol..

[19] Russell D.W., Sambrook J. (2001). Molecular Cloning: A Laboratory Manual.

[20] Hong W., Yan S. (2012). Engineering *Streptomyces tenebrarius* to synthesize single component of carbamoyl tobramycin. Lett. Appl. Microbiol..

[21] Wan Y., Hong W., Shi X. (2016). Study on the function of *sisI*, a biosynthetic gene of Sisomicin. J. Yanbian Univ..

[22] Pharmacopoeia Commission of the Ministry of Health of the People’s Republic of China (2010). Pharmacopoeia of the People’s Republic of China.

[23] Park J.W., Hong J.S., Parajuli N., Koh H.S., Park S.R., Lee M.O., Lim S.K., Yoon Y.J. (2007). Analytical profiling of biosynthetic intermediates involved in the gentamicin pathway of *Micromonospora echinospora* by high-performance liquid chromatography using electrospray ionization mass spectrometric detection. Anal. Chem..

