# Supplementary material for: Exclusive Production of Gentamicin C1a from Micromonospora purpurea by Metabolic Engineering
Source: Antibiotics (Basel). 2019 Dec 14;8(4):267. doi: 10.3390/antibiotics8040267 (PMC6963548; doi:10.3390/antibiotics8040267)
Supplement: Supplementary file 1 [file antibiotics-08-00267-s001.pdf]

Supporting Information

Figure S1. Domain analysis of *genL* (*Orf6255*)

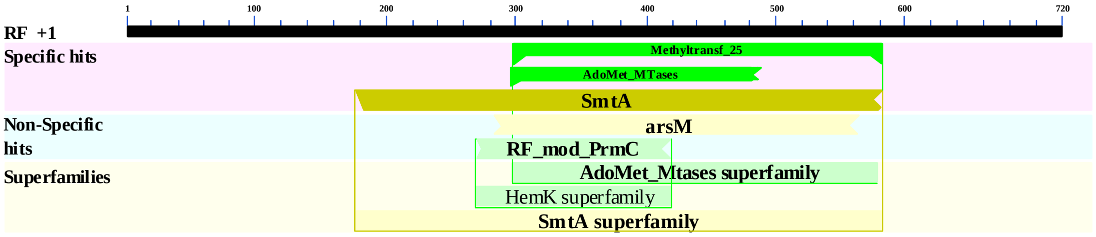

Table S1. List of GenL (Orf6255) conserved domain hits.

Name	Description	Interval (aa)	E-value
Methyltransf_25	Methyltransferase domain; This family appears to be a methyltransferase domain.	298-579	9.32e-11
AdoMet_MTases	S-adenosylmethionine-dependent methyltransferases (SAM or AdoMet-MTase)	295-486	3.53e-07
SmtA	SAM-dependent methyltransferase, Secondary metabolites biosynthesis, transport and catabolism,	175-579	1.52e-04

Figure S2. Thin layer chromatography analysis of metabolites of GbKL202

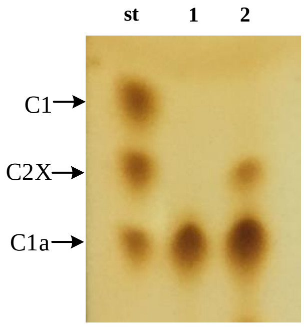

C2X=C2、C2a、C2b

Analysis of metabolites by TLC: Lane st: Standard gentamicin C complex including C1, C1a, C2, C2a and C2b (C2a and is C2 stereoisomer, they have the same Rf); Lane 1: Metabolites of GbkL202 mutant strain producing only C1a; Lane 2: Metabolites of GK1101 producing C2b and C1a.

Figure S3. Thin layer chromatography analysis of metabolites from GbL202

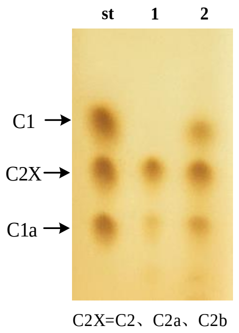

Analysis of metabolites by TLC: Lane st: Standard gentamicin C complex including C1, C1a, C2, C2a and C2b (C2a and is C2 stereoisomer, they have the same Rf); Lane 1: Metabolites of GbL202; Lane 2: Metabolites of wild type strain *M.purpurea* Gb1008 producing C1, C2, C2a, C2b and C1a.

Figure S4. Construction of GbHL202 (*genL* anaplerosis of GbL202) and Confirmation

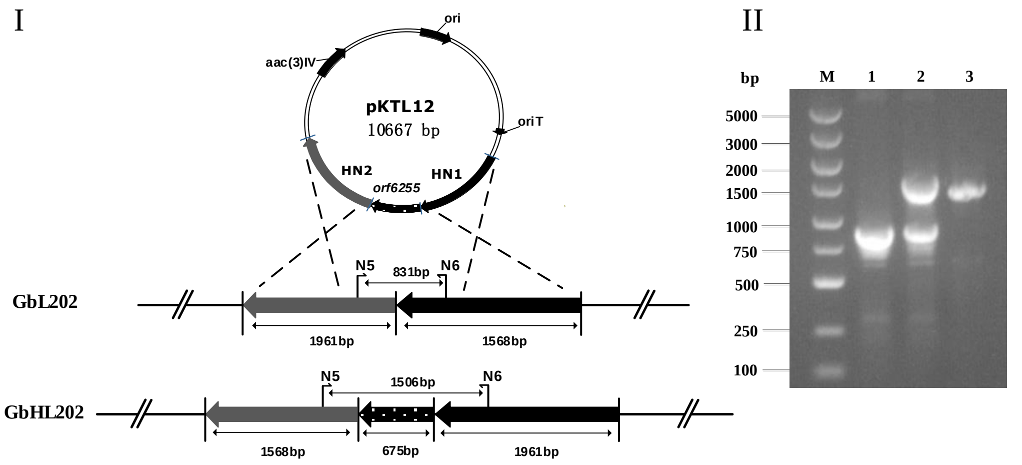

(I) Schematic presentation of the in-frame deletion; (II) DNA gel results. PCR analysis of the genomic DNA fragments from GbL202 and GbHL202 using primers N5 and N6. The gel results show: a 831 bp band (deleted 675 bp of *genL*) in GbL202 (lane 1); two bands at 1506 bp and 831 bp in single crossover mutant strain (lane 2); and a 1506 bp band (*genL* anaplerosis of GbL202) in GbHL202 (lane 3). The DL5000 DNA marker is in lane M.

Figure S5. MS and TLC analysis of metabolites from GbHL202

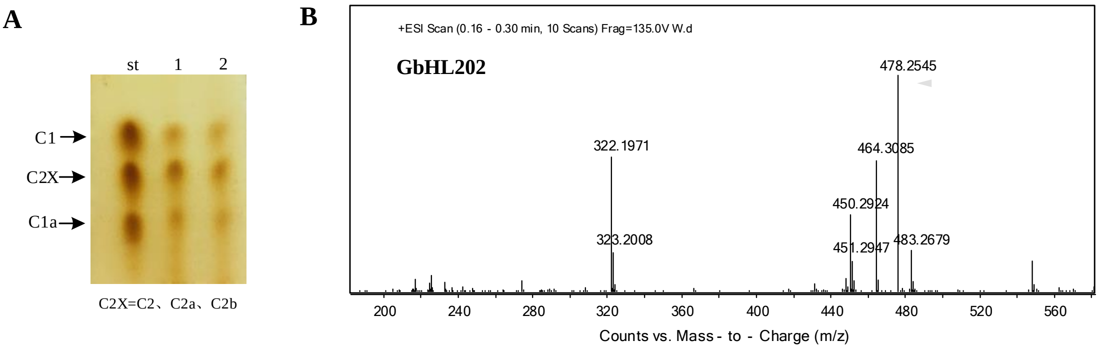

(A) TLC of metabolites from GbHL202 (*genL* anaplerosis of GbL202): Lane st: Standard gentamicin C complex including C1, C1a, C2, C2a and C2b (C2a and is C2 stereoisomer, they have the same Rf); Lane 1: Metabolites of GbHL202; Lane 2: Metabolites of the wild-type strain *M.purpurea* Gb1008 producing C1, C2, C2a, C2b and C1a.
(B) Mass spectrum analysis of metabolites from GbHL202.
